# Inhibitory Effects of* Glycyrrhiza glabra* and Its Major Constituent Glycyrrhizin on Inflammation-Associated Corneal Neovascularization

**DOI:** 10.1155/2018/8438101

**Published:** 2018-04-23

**Authors:** Syed Luqman Shah, Fazli Wahid, Noorullah Khan, Umar Farooq, Abdul Jabbar Shah, Shah Tareen, Fiaz Ahmad, Taous Khan

**Affiliations:** ^1^Department of Pharmacy, COMSATS Institute of Information Technology, Abbottabad 22060, Pakistan; ^2^Biotechnology Program, Department of Environmental Sciences, COMSATS Institute of Information Technology, Abbottabad 22060, Pakistan; ^3^Department of Pathology, Ayub Medical College, Abbottabad 22060, Pakistan

## Abstract

*Glycyrrhiza glabra *L. (Leguminosae) is widely used in folk medicines. Glycyrrhizin, an active compound of* G. glabra*, possesses anti-inflammatory activity. This study investigates the* G. glabra* methanol extract and glycyrrhizin for the treatment of corneal neovascularization (CNV).* G. glabra* was extracted in 70% aqueous methanol. Phytochemical tests, thin layer chromatography (TLC), and high performance liquid chromatography (HPLC) were used for the analysis of chemical composition. The topical solution of* G. glabra* methanol extract (2% w/v) and glycyrrhizin (1% w/v) was prepared in normal saline. After corneal burn (1 N NaOH), animals were left untreated for a week so that neovascularization appears in all groups. Treatments started on day 7 and continued for next 21 consecutive days. The animals were treated with 3 drops of various topical solutions thrice a day. Digital photograph analysis and histological studies were used for the evaluation of CNV. Phytochemical analysis of the* G. glabra* methanol extract showed the presence of saponins, phenols, carbohydrates, flavonoids, and proteins. TLC and HPLC confirmed the presence of glycyrrhizin. Photograph analysis of the extract and glycyrrhizin treated group showed a considerable decrease in CNV. Histological study of* G. glabra* and glycyrrhizin treated groups showed no blood vessels with properly arranged collagen fibers. This study showed that* G. glabra* and glycyrrhizin can be used for the treatment of CNV. Bioassay guided isolation can lead to preparation of ophthalmic solutions for the treatment of CNV.

## 1. Introduction

*Glycyrrhiza glabra* L. (Leguminosae) [1] is native to the Mediterranean region, central to Southern Russia and Asia, and now is widely cultivated throughout Europe and Middle East. Licorice has a high nutritional value and was used in foods since ancient times. In food, it is mainly used as a sweetening agent. It also has properties to inhibit the sensation of thirst [2]. Licorice oil is approved by the Food and Drug Administration (FDA) and has applications in various food products such as beverages, toothpaste, chewing gum, and cosmetics [3].* G. glabra* has been widely used in folk medicine for the treatment of different diseases [4].* G. glabra* leaves are used for the treatment of wounds [5] roots for diabetes, Graves' disease, and flatulence and stem for the treatment of tuberculosis [6]. It is also used as an aphrodisiac [7]. The methanol extract of the aerial parts of* G. glabra* showed antimicrobial activity against several bacterial species [8]. The aqueous methanol extract of* G. glabra* inhibited* in vitro *and* in vivo* proliferation of Ehrlich ascites tumor cells and showed antiangiogenic activity in* in vivo*, peritoneal, and chorioallantoic membrane assays [9]. Licorice also showed antiplatelet aggregation effects [10] and has also been used as a cough-relieving medicinal herb from ancient times [4]. Chinese licorice roots were found to inhibit the growth of* Plasmodium falciparum* and* Leishmania donovani* in* in vitro* studies [11]. Moreover, licorice has been reported for having antibacterial and antiviral activities [12].

*G*.* glabra* contains several chemical constituents, such as saponin, flavonoids, isoflavonoids, stilbenoids, and coumarins. The active constituents in saponins are glycyrrhizin, liquiritic acid, and glycyrretol. In flavonoids, the active constituents include liquirtin, liquiritigenin, and neoliquiritin. In isoflavonoids, they are glabridin, glabrone, glyzarin, and galbrene. The active constituents in coumarins are liqcoumarin and umbelliferone [13]. Finally, in stilbenoids, the active constituent is dihydrostilbenes [14]. Glycyrrhizin is a potassium and calcium salt of glycyrrhizinic acid. It is a saponin glycoside which upon hydrolysis yields glycyrrhetinic acid [15]. Glycyrrhizin, also known as glycyrrhizic acid, is the major (10 to 25%) active constituent of* G. glabra* root extract [16]. Glycyrrhizin helps in inhibition of lung cancer and fibro sarcomas [17]. In Japan, it has been used for the treatment of Hepatitis C for more than 60 years [18]. Furthermore, glycyrrhizin exhibited proapoptotic properties in a hepatocyte model of cholestatic liver injury. Glycyrrhetinic acid was found to be a potent inhibitor of bile acid-induced apoptosis and necrosis [19]. Members of the flavonoids like isoliquiritigenin and licochalcone have been found to possess antioxidant, antitumor, anti-inflammatory, and antiangiogenic activities [20, 21].

The cornea is a transparent, avascular, and outer tissue of the eye. Its transparency is necessary for clarity of vision. In normal conditions, corneal avascularity is maintained by a balance between angiogenic and antiangiogenic factors. The shift of this balance towards angiogenic factors causes corneal neovascularization (CNV) [22]. CNV is a pathologic condition in which the transparency of cornea is lost due to the ingrowth of new blood vessel from the limbus region of the eye. Development of CNV leads to a reduction in corneal transparency and consequently causes visual impairment usually loss of the central vision or can even result in blindness [23].

Although the exact cause of CNV is not yet completely identified, several pathological conditions such as inflammation, infection, degeneration, and traumatic disorders may induce CNV. Uses of contact lenses also induce hypoxia, which may ultimately leads to CNV. Among these factors, infectious diseases of the cornea are the most important cause of CNV [24]. Currently, the treatment options for CNV include medications like nonsteroidal anti-inflammatory drugs (NSAIDs), steroids, and cyclosporine [25, 26], laser therapies like thermal argon laser photocoagulation [27], amniotic membrane transplantation, and limbal transplantation [28]. However, all the available treatments have disadvantages such as high cost, low efficacy, and severe side effects.

There is a dire need to find new and alternative treatment for CNV. In the past, it was observed that extracts and compounds with anti-inflammatory and antiangiogenic activities have the potential to treat CNV. Based on the ethnopharmacological and other pieces of scientific information about anti-inflammatory and antiangiogenic activities of* G*.* glabra *extract and its major chemical constituent, glycyrrhizin, the current study was carried out to assess their potential for the treatment of CNV. It was found that the* G*.* glabra *extract effectively halted CNV but glycyrrhizin was found to be relatively less effective in stopping the development of CNV in animal model.

## 2. Materials and Methods

### 2.1. Plant Material

*G. glabra* roots were purchased in June 2015 from an authentic herbal store in Abbottabad, Pakistan. The identification of the specimen (voucher number gg-09-R/15) was confirmed by Dr. Abdul Nazir, Assistant Professor, COMSATS Institute of Information Technology, Abbottabad, Pakistan, and plant specimen was deposited to Department of Pharmacy, COMSATS Institute of Information Technology, Abbottabad, Pakistan.

### 2.2. Chemicals

Glycyrrhizin (purity ≥ 75%) was purchased from Sigma Aldrich, USA. Ketamine HCl (Indus Pharma, Pakistan), xylazine HCl (FARVET, Peru), proparacaine HCl (Alcon Laboratories, Inc., USA), and normal saline (Otsuka Pakistan Ltd.) were purchased from a local pharmacy store.

### 2.3. Processing and Extraction

The dried plant material was ground and extracted by soaking powdered material (745 g) in 70% aqueous methanol (3 L) at ambient temperature. The mixture was occasionally stirred with a stainless-steel rod for two weeks to get the maximum extract. The extract was filtered through a muslin cloth followed by Whatman filter paper number 42 (125 mm). The extract was concentrated using a vacuum rotary evaporator (Yamato Rotary Evaporator, RE 801; South Korea) with the water bath set at 40°C. The final percentage yield of the extract was 12.2%.

### 2.4. Phytochemical Analysis and Fingerprinting

The crude extract was subjected to preliminary phytochemical analysis for all major chemical constituents using standard chemical tests [29, 30]. For alkaloids, the extract (500 mg) was shaken with 5 mL of 1% HCl and heated gently for 1 min using a water bath. Next, 1 mL of this solution was taken and 0.5 mL of Wagner's reagent was added. The development of turbidity or precipitates indicated the presence of alkaloids. For steroids, Salkwoski's test was used in which 2 mL chloroform was added to dissolve 100 mg of extract in a test tube. Then, 2 mL of the concentrated H_2_SO_4_ was carefully added to form the lower layer. A green color formed on the upper layer indicating the presence of steroids. For saponins (froth test), 3 mg of extract was dissolved in 10 mL distilled water and vigorously shaken in a test tube and allowed to stand for 1 min. The development of consistent froth indicated the presence of saponins. For flavonoids (lead acetate test), 1 mL lead acetate solution (5%) was added to 1 mg of the plant extract in a test tube. The mixture was allowed to stand undisturbed. Formation of yellow colored precipitates indicated the presence of flavonoids. For phenols (ferric chloride test), 2 mg extract was taken in a test tube and 3 drops of 10% ferric chloride were added. The appearance of a bluish-black color indicated the presence of phenols. For glycosides (nitroprusside test), methanol extract was added with a few drops of 10% sodium hydroxide and then sodium nitroprusside was added to the above solution. The appearance of a blue color indicated the presence of glycosides in the extract. For reducing sugars, Fehling's solution (A and B) was added to aqueous methanol extract (100 mg/mL) in a test tube. The resulting solution was heated on a water bath for 10 min. Formation of a red-orange precipitate was a positive indication for the presence of reducing sugars. For the detection of proteins, the plant extract was treated with few drops of concentrated nitric acid. Formation of a yellow color indicated the presence of proteins.

Thin layer chromatography (TLC) was used for the identification of various compounds, especially the major chemical constituent, glycyrrhizin. The extract and glycyrrhizin were applied to TLC plates coated with silica gel (60 F254) and developed in n-butanol, acetic acid, and distilled water (12 : 3 : 5) as the mobile phase. Ceric sulfate was used as the spraying reagent for the visualization of compounds on TLC plates.

High performance liquid chromatography (HPLC) (Perkin Elmer, Series 200 Auto sampler) was used for the analysis of extract and glycyrrhizin. The following conditions were applied during the HPLC fingerprinting: UV-Vis detector (200–700 nm), column (C18) (5 *μ*m, 150 mm × 4.6 mm), mobile phase which was acetonitrile and water in the ratio of 10 : 90, injection volume which was 20 *μ*L, flow rate which was 1 mL/min, and membrane filter of 0.45 *μ*m were used. The compounds were detected at a wavelength of 254 nm. For the* G. glabra*, 100 mg of the extract was taken in 25 mL of 70% aqueous methanol in a volumetric flask (50 mL) and sonicated for 50 min at ambient temperature. Glycyrrhizin standard solution was prepared by dissolving 5 mg glycyrrhizin in methanol and final volume was made 10 mL with further addition of methanol. The stock solutions of* G. glabra *extract and glycyrrhizin were filtered and degassed ultrasonically before analysis.

### 2.5. Animals

Rabbits of either gender (1.5–2 kg) were used as the animal model in the current study. These were purchased from the local market in Abbottabad City. The experimental protocol was approved under the regulations of CIIT Abbottabad Ethical committee for the animals with approval number PHM.Eth/SP.14-714-CIIT-ATD on July 11, 2015, which follows all recommendations of the NIH Animal Ethical Guideline (1986). Rabbits were randomly separated into four groups with a minimum of five animals per group. After that, each rabbit was further tagged on the ear within the groups to guarantee their partition during the whole experimental procedure. Animals were placed in the standard condition and given free access to food and water.

### 2.6. Induction of Corneal Burn in Rabbit's Eye

For the induction of CNV, the right eyes of all the experimental animals were damaged by alkali burn (1 N sodium hydroxide solution) using the reported protocol [31]. Briefly, the experimental animals were starved for 12 h before starting the experiment. The animals were anesthetized using an intramuscular injection of ketamine HCl (50 mg/kg) and xylazine (5 mg/kg) in combination. After that, the right eye of the rabbit was opened with a wire speculum so that alkali burn could be induced properly. Approximately, 2 min before the burn, a few drops of proparacaine HCl were instilled into the right eye of each rabbit to minimize the irritation in the cornea. A paper punch was used to produce a 7 mm disc from Whatman filter paper. The filter paper discs were gently dipped into 1 N sodium hydroxide solution for about 90 s and subsequently placed centrally on cornea for 2 min to induce severe burns. The eyes were washed with normal saline for moistening and to obtain sterility. After the corneal burn, the rabbits were kept for a week for the development of CNV without further treatments.

### 2.7. Preparations of Eye Drops and Treatment Protocols

The topical solution of* G. glabra *extract (2% w/v) was prepared in normal saline using 10% dimethyl sulfoxide (DMSO) and a few drops of Tween-80 and mixed using vortex mixer. Similarly, glycyrrhizin (1% w/v) solution was prepared in normal saline. For preparation of the vehicle, dimethyl sulfoxide (DMSO) (10% v/v) and few drops of Tween-80 were mixed in normal saline. Dexamethasone (0.1%) (ALCON-COUVREUR, Belgium) was obtained from the local market and used as a positive control. All topical solutions were packed in special droppers purchased from the local market and stored at 4°C. Treatments were started on the 7th day of the corneal burns and continued for next 21 consecutive days. The eyes of different animal groups were topically treated with 3 drops of various solutions three times a day. The first group received treatment of* G. glabra* extract while second group was treated with glycyrrhizin. The third group was given the vehicle and the fourth group treated with dexamethasone (0.1%) was considered as a positive control.

### 2.8. Microscopic and Photographic Analysis

The progression of CNV in all groups was periodically monitored through a slit-lamp microscope (Olympus-CX21) with a manually attached artificial light source. The photographs were taken using a digital still camera (DSC-W70, Sony, Japan) with magnification ×12, 85 mm lens distance, 100% zoom rate, and 29 cm shooting distance. The photographs were taken on days 1, 7, 14, 21, and 28 and stored in the computer for further use.

### 2.9. Histological Studies of Corneal Tissues

On the last day of the experiment, the animals were sacrificed with cervical dislocation. Whole eyes were extracted and preserved in 10% neutral formalin. After preservations, corneal tissue samples were removed from the whole eye using surgical blades. The tissues were then transferred to perforated jars (capsule) using forceps. These tissues were immersed in formalin (15%) and then in alcoholic formaldehyde saline (15%) for 3 h each. The tissues were then dehydrated using ascending grades of alcohol (ethanol). During this process tissues were treated with 70% and 80% alcohol for 2 h each and finally with 100% alcohol for 6 h. In the next step, the samples were cleared in pure xylene for 6 h (3 changes every 2 hours in different jars). The tissues were then embedded in paraffin wax using an automatic hot cabin having a temperature range of 58 ± 5°C. The tissues were dipped in paraffin wax for 4 h (2 h in 2 shifts and in different jars). After this, sections of 4 *μ*m of corneal tissues were obtained using a rotatory microtome (Thermo Fisher Scientific, Germany). For proper fixation, egg albumin drops were placed over slides and a single tissue section was mounted on to it. Then the slides were placed for 2 h in a precision mechanical conviction incubator (slide warmer) (Model 4EM Cat. number 31574). Histological haematoxylin & eosin (H&E) staining was performed through different steps including dewaxing, hydration, haematoxylin staining, decolourization (eosin staining), and dehydration.

## 3. Results

### 3.1. Phytochemical Analysis of* G. glabra*

Phytochemical analysis of the* G. glabra* extract was performed to find out the presence of the major classes of phytochemical constituents. The results revealed the presence of flavonoids, carbohydrate, protein, saponins, and phenols as shown in Table 1. The presence of alkaloids, phytosterol, and glycosides were not confirmed in the current investigation (Table 1).

**Figure 1:**
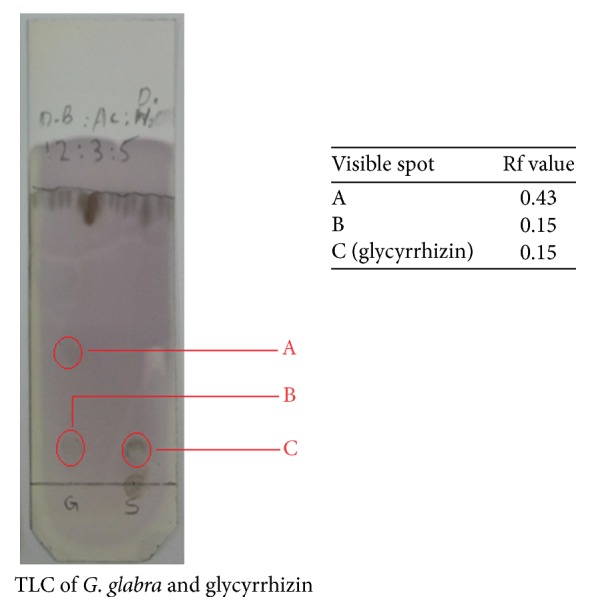
TLC profile for the analysis of* G. glabra* extract and glycyrrhizin using silica gel coated TLC plates and n-butanol : acetic acid : water (12 : 3 : 5) as the mobile phase.

The results obtained from chemical tests were further confirmed through TLC analysis. The extract was standardized with reference to its major chemical constituent, glycyrrhizin. The results have been shown in Figure 1. The retention factor (Rf) values of the constituents of the extract were compared with glycyrrhizin. As shown in Figure 1, the Rf value for the visible spot A in* G. glabra* extract was 0.43 while for B was 0.15. The Rf value for glycyrrhizin (visible spot C) was also found to be 0.15. On the basis of these results, it may be confirmed that the glycyrrhizin was present in the extract as one of the major compounds.

**Figure 2:**
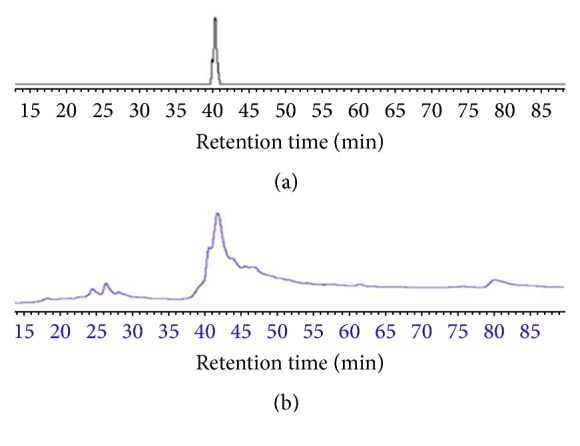
HPLC chromatogram of (a) glycyrrhizin and (b)* G. glabra* extract. HPLC conditions were wavelength (254 nm), mobile phase (acetonitrile : water), injection volume (20 *μ*L), and flow rate (1 mL/min).

HPLC analysis of the crude extract derived from* G. glabra* and glycyrrhizin was carried out to get the major peaks for various chemical constituents in extracts and to confirm the presence of the major compound (glycyrrhizin). The resulting chromatogram displayed various peaks at different retention times for the* G. glabra *extract as shown in Figure 2(b). The chromatogram also showed a peak at a retention time of 40 min for glycyrrhizin as displayed in Figure 2(a). Similar to glycyrrhizin, a peak was also observed in the chromatogram for the extract at the same retention time (40 min) as shown in Figure 2(a). The results confirmed the presence of glycyrrhizin as the major component in the extract of the* G. glabra*.

### 3.2. CNV Analysis of* G. glabra* Extract, Glycyrrhizin, Vehicle, and Positive Control Treated Groups

**Figure 3:**
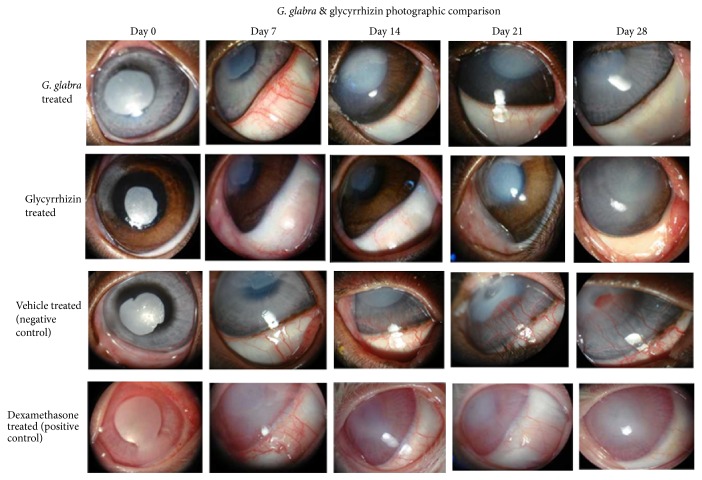
Representative photographs of CNV in* G. glabra *extract, glycyrrhizin, vehicle, and dexamethasone (positive control) treated group on days 0, 7, 14, 21, and 28. There is a gradual decrease in diameter and thickness of NV, and it almost vanished on the last day of experiment. The glycyrrhizin treated group also showed decrease in diameter and thickness of NV but the activity was lesser than the* G. glabra *extract.

To observe the efficacy of the* G. glabra* extract and glycyrrhizin treatment, the progression of CNV was monitored under microscopic and through photographic analysis on different treatment days. The photographs taken on different treatment days have been shown in Figure 3 for extract and vehicle treated groups. It was observed under the microscope and can also be seen in photographs that, even in the first week of treatment, the thickness of the neovessel (NV) increases in the extract treated group. However, a faster decrease was seen in the vessel's thickness after the second week (Figure 3) and continued until the end of the experiment. The obtained results showed that the* G. glabra* extract almost completely vanished CNV on day 28 of the experiment as compared to the vehicle control group (Figure 3). The dexamethasone (positive control) treated group (Figure 3) also showed the same trend as that for the* G. glabra* extract treated group. It was also revealed from the microscopic observation as well as photographic analysis that glycyrrhizin also showed positive effects in blocking CNV. When the eyes were analyzed using a high power resolution microscope, it was seen that there is a gradual fading of blood vessel and resumption to normal shape on the last day of treatment (Figure 3) with the presence of only few NV. These results showed that anti-CNV activity of glycyrrhizin was slightly lower than that of the crude extract.

### 3.3. Histological Analysis of* G. glabra* and Glycyrrhizin Treated Cornea in Comparison with Vehicle and Positive Control Group

**Figure 4:**
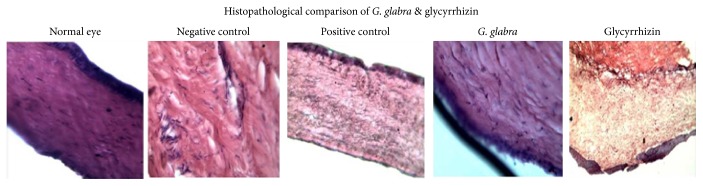
Histological microphotograph of* G. glabra* extract and glycyrrhizin treated group in comparison to vehicle control and positive control.

The histological analysis was performed to know the presence of inflammation, corneal fiber recovery, and the existence of blood vessels. The H&E stained representative microphotographs have been shown in Figure 4. The histology of the left eye of each animal was considered as a reference tissue as no alkali burns were induced and were kept in normal conditions. In the reference cornea, there was no epithelial and NV growth as well as no changes in morphology as shown in Figure 4. In the vehicle control group (Figure 4), there were extensive blood vessels and collagen disruption, which showed the development of CNV. On the other hand, the histology of the* G. glabra* extracts treated cornea showed that the corneal region has almost recovered to normal with blood vessels nearly diminished and collagens in normal shape. The histological analysis of the glycyrrhizin treated group (Figure 4) showed that although the corneal region is mostly recovered to its normal shape, there are some indications that damage is still present. There were some signs for epithelial hypertrophy and fewer blood vessels were also observed. These results showed that glycyrrhizin is effective in inhibiting CNV but to a lesser extent than* G. glabra* extract. In comparison, the H&E stained slides microphotograph of the dexamethasone treated (Figure 4) cornea showed reduction of epithelial growth and blood vessel formation in the corneal region but collagen fibers were somehow in a destructed state and haphazardly arranged.

## 4. Discussion

CNV is one of the major causes of blindness around the world. It has been estimated that about 4.14% of world population is affected by this disease [32]. The main molecular cause for CNV is the imbalance between angiogenic and antiangiogenic factors. Among these factors, vascular endothelial growth factor (VEGF) promotes the vascular endothelial cell propagation, passage, and tube formation [32]. So, there is a possibility of treating CNV with anti-VEGF agents; however, it cannot be completely treated merely with anti-VEGF therapy as there are also some additional regulators of angiogenesis [33]. Moreover, inflammation and angiogenesis are interdependent processes and thus the anti-VEGF treatment may not be successful [34, 35]. In the past, it was observed that extracts and compounds having antitumor and anti-inflammatory activity are helpful in the inhibition of NV and CNV [36, 37]. The crude extract of* G. glabra* has shown an anti-inflammatory effect while the aqueous extract of* G. glabra* has inhibited angiogenesis in* in vivo* assays [9]. Therefore, it was hypothesized that the* G. glabra* extract and its major chemical constituent, glycyrrhizin, may have the ability to control CNV and thus the current study was undertaken.

It is important to know the chemical composition of any extracts before evaluating it for biological activities. Therefore, phytochemical analysis of the* G. glabra* extract was carried out, which revealed that it contains important chemical constituents like flavonoids, carbohydrate, protein, saponins, and phenols. Previous studies reported the presence of saponin, flavonoids, alkaloids, terpenoids, tannins, and glycosides, but carbohydrates, proteins, phlobatannins, phenolic compounds, and anthraquinones were not detected [38]. Another study showed the presence of carbohydrates, phenolic compounds, and proteins along with other constituents in the* G. glabra *extract [39]. The reasons for these differences may be season and age of the collected plants.

TLC and HPLC confirmed the presence of glycyrrhizin in the crude extract. TLC and HPLC chromatograms of the extract revealed a compound at Rf value (0.15) and retention time (about 40 min) as that of standard glycyrrhizin. These results are a little deviation from previous observations in which the standard glycyrrhizin was spotted at 0.22 [40]. The main reason for this might be the use of different reagent and conditions in the previous and present study. In the previous study, chloroform, methanol, and water were used as mobile phase and anisaldehyde-sulfuric acid as spraying reagent while in present study n-butanol, acetic acid, and distilled water were used as the mobile phase and ceric sulfate as spraying reagent.

Glycyrrhizin, one of the major components of* G. glabra*, has been reported to possess anti-inflammatory and anticancer activities [41]. The results of the current study revealed that the* G. glabra* extract was successful in inhibiting CNV as NV were almost diminished after 21 days of treatment (Figure 3). Being the major compound, it was thought that the inhibition of CNV might be due to glycyrrhizin. The anti-CNV analysis of glycyrrhizin showed some positive effects on the inhibition of CNV but was unable to completely diminish blood vessels in the corneal region (Figure 3). Glycyrrhizin is a triterpenoid glycoside (saponin) with glycyrrhetinic acid. Previously, it was reported that glycyrrhetinic acid has direct effect on mineralocorticoid receptors and produces inflammatory like effects [42], which may be one of the reasons for the lower effects of glycyrrhizin. This study suggests that along with glycyrrhizin some other compounds may be responsible for the inhibition of CNV in the* G. glabra *extract.

It was also important to study the effects of the* G. glabra *extract and glycyrrhizin on microscopic anatomy (microanatomy) of the corneal tissue. Therefore, histological analysis was performed and its results confirmed the anti-CNV effects of the* G. glabra* extract. Moreover, there was no clear sign of toxicity as the collagen fibers were arranged in an array in the* G. glabra *extract treated animal cornea. The microscopic anatomy of the* G. glabra *extract treated cornea was very similar to that of the normal control. On the other hand, there were no signs of blood vessels in dexamethasone (positive control) treated cornea, but the collagen fibers were still in disarray which showed that dexamethasone may have some toxic effects on the cornea. Although, previous studies also reported toxic effects of dexamethasone [43], further research is needed to confirm this phenomenon at the microanatomical level. It is also important to mention that although glycyrrhizin was largely successful to control CNV, it was unable to completely recover the alkali damaged cornea and some blood vessels were also observed.

## 5. Conclusions

It may be concluded from the results that the ophthalmic drops of the crude extract of* G. glabra *inhibited the growth of vessels in the corneal region, which indicates that it would be effective in the treatment of CNV. Moreover, the major compound of* G. glabra*, glycyrrhizin, was unable to completely impede the CNV. This means that some other constitute(s) may be also responsible, along with glycyrrhizin, for anti-CNV activity of the extract. For this purpose, further research is required on the bioassay guided isolation for the identification of the main component(s) responsible for the inhibition of CNV. Furthermore, molecular studies will also be helpful to find out the exact molecular mechanism of the* G. glabra *extract in treatment of CNV.

## Figures and Tables

**Table 1:** Phytochemical analysis of *G. glabra* extract showed the presence of saponins, flavonoids, phenols, carbohydrates, and proteins.

Phytochemical components	Chemical test	Presence
Alkaloids	Wagner's test	−
Phytosterol test	Salkowski's test	−
Saponins	Foam/Froth test	+
Flavonoids	Alkaline reagent test	+
Phenols	Ferric chloride test	+
Glycosides	Nitroprusside test	−
Carbohydrates	Fehling's test	+
Proteins	Xanthoproteic test	+

+ = evidence of phytochemicals; − = no evidence of phytochemicals.
